# Supplementary material for: Quantitative genetic analysis of respiratory function and related traits in Bulldogs, French Bulldogs and Pugs
Source: PLoS One. 2026 May 13;21(5):e0348023. doi: 10.1371/journal.pone.0348023 (PMC13170967; doi:10.1371/journal.pone.0348023)
Supplement: S1 File — (DOCX) [file pone.0348023.s001.docx]

**Supplement 1 – Respiratory Function Grading Scheme (RFGS) Details**

The RFGS was introduced by the RKC, in collaboration with the University of Cambridge, in 2019. The scheme provides a standardised, non-invasive assessment of BOAS severity in Bulldogs, French Bulldogs, and Pugs under field conditions. The assessment consists of a three-minute exercise test at ~3-4 mph, with respiratory function evaluated before and immediately after exercise by visual observation and pharyngolaryngeal auscultation. Parameters recorded include stertor and stridor, inspiratory effort, and clinical signs such as dyspnoea, cyanosis, or syncope. The most severe observation from the pre- and post-exercise assessments is used to determine the final functional grade (1,2). Functional grades range from 0 to 3, with grades 0–1 considered clinically unaffected and grades 2–3 indicating disease with increasing severity. Over time, the protocol has been refined in response to field data; notably, stridor was removed from the Bulldog and French Bulldog protocols in February 2023 due to misclassification of nasal versus laryngeal stridor but was reinstated in November 2024 following reports of clinically significant laryngeal stridor.

Current breeding guidance associated with the scheme is presented in a traffic-light matrix categorising mating pairs as green, amber, or red to indicate low, moderate, and high risk of producing BOAS-affected offspring, respectively. Pairings between grade 0 and grade 1 dogs are designated green, those involving a grade 2 dog with grades 0, 1, or 2 are amber, and any pairing including a grade 3 dog is red and not recommended. When the scheme was first introduced, matings between grade 2 and grade 0 dogs were classified as green but were reclassified as amber in June 2024 following review of scheme data and welfare considerations (3)

Assigned functional grades are valid for 2 years, with re-testing recommended every 2 years or until a dog is retired from breeding. RFG results are recorded in the RKC database and displayed publicly on the RKC website, with assessments undertaken by approved veterinary assessors across the UK.

In addition to national implementation, the RFGS has been licensed to 21 international organisations for integration into their national BOAS screening and breeding programmes, highlighting the scheme’s applicability and standardisation potential beyond the UK (FCI, 2020).

1. Liu NC, Sargan DR, Adams VJ, Ladlow JF. Characterisation of Brachycephalic Obstructive Airway Syndrome in French Bulldogs Using Whole-Body Barometric Plethysmography. Rosenfield CS, editor. PLOS ONE. 2015 June 16;10(6):e0130741.

2. Riggs J, Liu N, Sutton DR, Sargan D, Ladlow JF. Validation of exercise testing and laryngeal auscultation for grading brachycephalic obstructive airway syndrome in pugs, French bulldogs, and English bulldogs by using whole‐body barometric plethysmography. Vet Surg. 2019 May;48(4):488–96.

3. The Royal Kennel Club. RFGS Breeding Recommendations [Internet]. 2024 [cited 2025 Oct 21]. Available from: https://www.thekennelclub.org.uk/health-and-dog-care/health/getting-started-with-health-testing-and-screening/respiratory-function-grading-scheme/#:~:text=them%20for%20breeding.-,Breeding%20advice,-For%20breeds%20where

4. FCI, 2020. Brachycephalic breeds’ health: agreement between the Kennel Club (UK) and the FCI. https://www.fci.be/en/Brachycephalic-breeds-health-agreement-between-the-Kennel-Club-UK-and-the-FCI-3556.html
