# Supplementary material for: Quantitative genetic analysis of respiratory function and related traits in Bulldogs, French Bulldogs and Pugs
Source: PLoS One. 2026 May 13;21(5):e0348023. doi: 10.1371/journal.pone.0348023 (PMC13170967; doi:10.1371/journal.pone.0348023)
Supplement: S2 File — (DOCX) [file pone.0348023.s002.docx]

To obtain a preliminary estimate of the heritability of RFGS grade, we conducted a parent–offspring regression. Heritability was estimated as the regression coefficient (b) in the model

$$y=a+bX+e$$

where y is the offspring phenotype, a is the intercept, X is the mean parental phenotype, and e is the residual error.

For dogs with multiple grade records, the highest (worst) grade was used. Regressions were performed on all available parent–offspring trios combined and separately by breed. Results are presented in Table S3.1.

Table 1 Parent-offspring regression estimates of heritability for RFGS grade

|  | Number of trios | $h^{2} (SE)$ | p-value |
| --- | --- | --- | --- |
| Bulldog | 275 | 0.21 (0.11) | 0.06 |
| French Bulldog | 270 | 0.33 (0.09) | <0.001 |
| Pug | 157 | 0.38 (0.10) | <0.001 |
| ALL | 702 | 0.31 (0.06) | <0.001 |

The estimates indicate moderate heritability in French Bulldog, Pug, and in the combined analysis. In Bulldog, the estimate did not differ significantly from zero despite the largest sample size.
