# Supplementary figures and images for: Quantitative genetic analysis of respiratory function and related traits in Bulldogs, French Bulldogs and Pugs
Source: PLoS One. 2026 May 13;21(5):e0348023. doi: 10.1371/journal.pone.0348023 (PMC13170967; doi:10.1371/journal.pone.0348023)

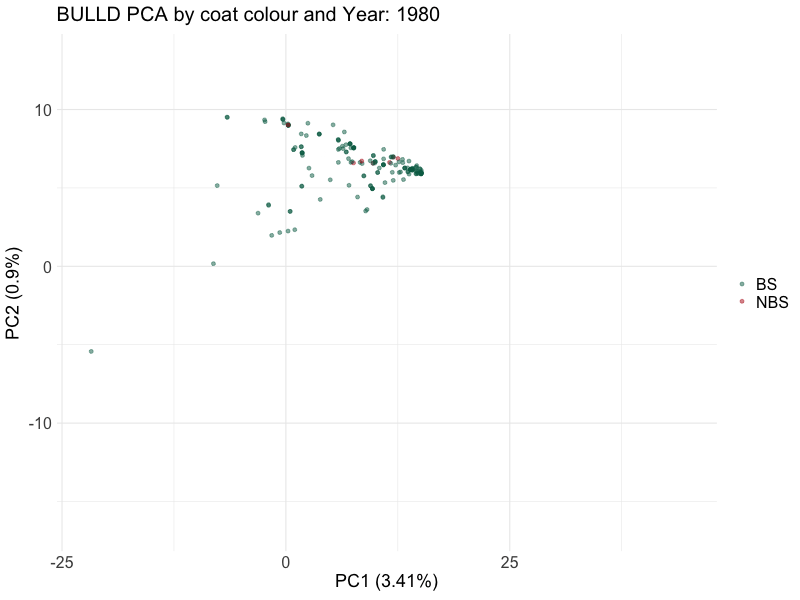

Supplement: S1 Fig — (GIF) [file pone.0348023.s003.gif]

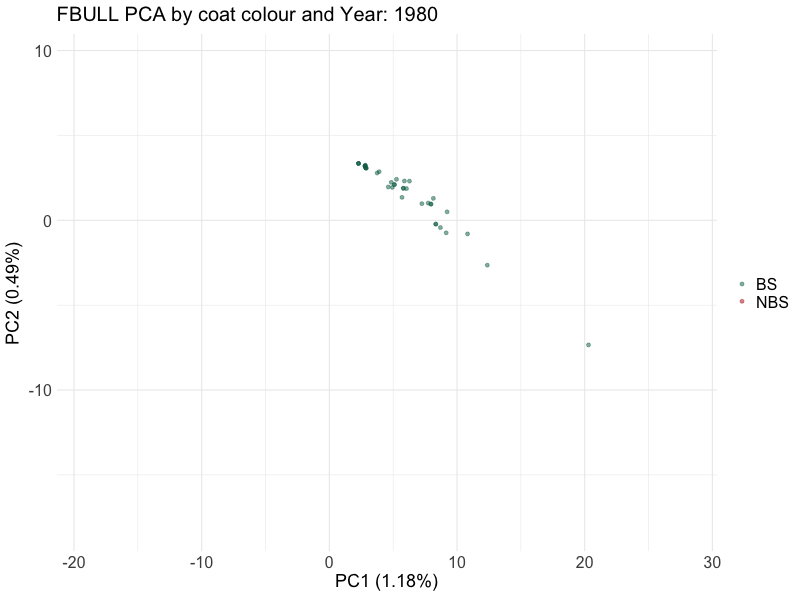

Supplement: S2 Fig — (GIF) [file pone.0348023.s004.gif]

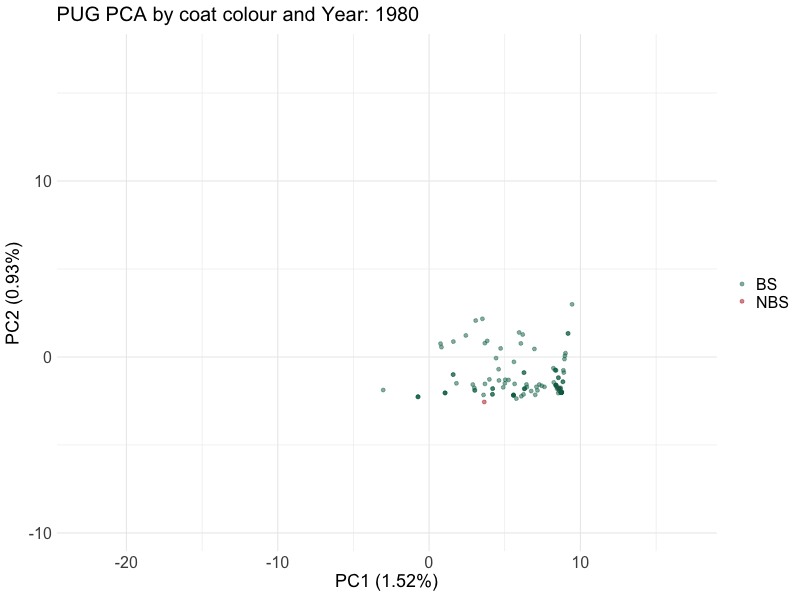

Supplement: S3 Fig — (GIF) [file pone.0348023.s005.gif]
