# Supplementary material for: Quantitative genetic analysis of respiratory function and related traits in Bulldogs, French Bulldogs and Pugs
Source: PLoS One. 2026 May 13;21(5):e0348023. doi: 10.1371/journal.pone.0348023 (PMC13170967; doi:10.1371/journal.pone.0348023)
Supplement: S2 Table — (DOCX) [file pone.0348023.s007.docx]

Table S6 Results of multi-variate analyses. Diagonal cells are not applicable and are therefore left empty and shaded grey. Above-diagonal cells show assessor effect correlations, below-diagonal cells show residual correlations.. All models included sex, linear age, age splines, and coefficient of inbreeding. Colour group was fitted in Bulldog and Pug only. BCS was included as a covariate for grade and BW in Bulldog and Pug, and as a separate trait in French Bulldog. Obesity was fitted for all traits except grade in Pug. Year of test and date of birth were fitted to BW and STEN grade in Bulldog and French Bulldog, and date of birth was also fitted to STEN grade in Pug. Protocol was fitted only for STEN grade in Bulldog, and year of birth only for BW in Bulldog. Significance is indicated by p < 0.05 (*), p < 0.01 (**), p < 0.001 (***).

|  | BULLD | | | FBULL | | | | PUG | | |
| --- | --- | --- | --- | --- | --- | --- | --- | --- | --- | --- |
|  | Grade | BW | STEN | Grade | BW | STEN | BCS | Grade | BW | STEN |
| Grade |  | -0.04 (0.33) | 0.66 (0.19) ** |  | NA | 0.15 (0.27) | -0.22 (0.23) |  | 0.52 (0.29) | 0.68 (0.20) * |
| BW | 0.18 (0.10) * |  | -0.13 (0.31) | 0.14 (0.09) |  | NA | NA | -0.14 (0.14) |  | 0.60 (0.23) * |
| STEN | 0.17 (0.07) ** | 0.00 (0.09) |  | 0.26 (0.06) *** | 0.10 (0.09) |  | 0.09 (0.24) | 015 (0.09) | -0.09 (0.14) |  |
| BCS |  |  |  | 0.27 (0.06) *** | 0.49 (0.07) *** | 0.16 (0.06) ** |  |  |  |  |
